# Evaluation of Electromyographic Activity of Masticatory Muscles in Adults with Posterior Crossbite

**DOI:** 10.1155/2022/4552674

**Published:** 2022-03-12

**Authors:** Luiz Makito Osawa Gutierrez, Melissa Coradini Quatrin, Chiarella Sforza, Rafael Reimann Baptista, Eduardo Martinelli Santayana de Lima

**Affiliations:** ^1^Post-Graduate Program in Dentistry, School of Health and Life Sciences, Pontifical Catholic University of Rio Grande do Sul, PUCRS, Porto Alegre, RS, Brazil; ^2^Human Anatomy, Medical School, Università degli Studi di Milano, Milan, Italy

## Abstract

**Introduction:**

There is evidence that patients with posterior crossbite (PXB) have neuromuscular changes in the masticatory muscles. However, up to the present time, the relationship among these changes on the electromyographic activity of the masticatory muscles is still unclear.

**Objective:**

To systematic review the available literature on the electromyographic activity of masticatory muscles in adults with PXB.

**Methods:**

Between August 22 and September 9, 2020, we searched the following seven electronic databases: PubMed, EMBASE, Web of Science, Cochrane Library, SciELO, LILACS, and Scopus. No restrictions were applied regarding the language and year of publication. This systematic review was registered in the Prospective Register of Systematic Reviews (PROSPERO - CRD42020205057) database and conducted using the Preferred Reporting Items for Systematic Reviews and Meta-Analysis (PRISMA) guidelines. After data selection and extraction, the methodological quality of the selected studies was conducted independently by two reviewers, using two different evaluation tools.

**Results:**

6957 records were initially located after the search process. In the end, eight papers were selected. Most studies were classified as having average to low methodological quality and moderate to high risk of bias. Based on the available evidence, adult patients with PXB have electromyographic activity changes in the masticatory muscles when compared with individuals without PXB. Moreover, adult patients with unilateral posterior crossbite (UPXB) have asymmetrical electromyographic activity when the crossbite side is compared with the noncrossbite side.

**Conclusion:**

Despite the lack of studies with high methodological quality, electromyographic evaluation of masticatory muscles should be considered in the diagnosis and in the orthodontic treatment plan of patients with PXB. Prospective studies with a higher sample size and follow-up time, conducted using a rigorous scientific methodology, are necessary to reach a more reliable conclusion.

## 1. Introduction

Posterior crossbite (PXB) is defined as an inverted cross-sectional relationship between upper and lower teeth, when the vestibular cusps of premolars and upper molars occlude the occlusal fossae of the lower antagonist teeth [1]. The prevalence of PXB is from 8 to 22% in orthodontic patients in the mixed dentition stage and from 5 to 15% in the general population [2]. Within the demographic group of patients with PXB, 70 to 80% of the cases are unilateral posterior crossbite (UPXB) [3].

There is evidence suggesting that patients with PXB present changes in the neuromuscular activity of masticatory muscles [4–6]. This condition is associated with a functional mandibular lateral displacement caused by occlusal interferences, especially in patients with UPXB [4, 7]. This displacement changes the position of condyles in the glenoid cavity and can therefore corroborate the development of facial asymmetries and temporomandibular disorders (TMD) in adult patients [8].

The relation between muscular activity and skeletal morphology can influence the diagnosis of many pathologies [7]. In this sense, an accurate orthodontic diagnosis can be performed in patients with PXB through a morphological and functional evaluation [6, 9, 10].

The morphological evaluation reveals bone and dental discrepancies between the upper and lower arches, as well as facial aesthetic parameters. Radiographs, cephalometry, photographs, and cone beam computed tomography (CBCT) can be used for this purpose [9, 10]. CBCT and 3D cephalometry can be used for the analysis of the soft tissue facial profile measurements and for the study of skeletal discrepancy with respect to ideal values [9, 10].

The functional evaluation of patients with changes in masticatory muscle activity, on the other hand, can be performed by surface electromyography (sEMG) [6]. sEMG measures the sum of the action potentials sent by the central nervous system to the motor units [11]. sEMG is an excellent diagnostic tool and widely used in the muscular evaluation of patients with PXB because it is an objective and noninvasive technique [12]. With sEMG, it is possible to identify the intensity, frequency, and onset of muscle contraction between the right and left sides [5, 6, 13]. This information is important for determining muscle power, the specificity level of recruitment of motor units, and the degree of muscle balance and coordination between sides.

Balanced muscle activity is a fundamental condition for determining a balanced and functional occlusion [4]. To date, the available evidence on changes in the electromyographic activity of masticatory muscles in adults with PXB is not yet clear [4, 14]. Some studies point out that these patients present a decrease in electromyographic activity [15–17]. Others point to an increase in the activity of these muscles [7, 18]. There are also studies that did not find any differences [19]. Discordant data are also found regarding the side of the involvement in patients with UPXB. Some studies point out that these changes occur preferably on one side [15, 16, 18]. Others did not report differences [19].

In view of this discrepancy in the literature, the objective of the present study is to conduct a systematic review on the effects of PXB on the electromyographic activity of masticatory muscles in adults to respond to the following research problem: does PXB cause changes in the electromyographic activity of masticatory muscles in adults? Our hypothesis is that PXB changes the electromyographic activity of the masticatory muscles in an asymmetrical way, causing changes in the masticatory function.

## 2. Methods

### 2.1. Registration and Protocol

The protocol for this systematic review was registered in the Prospective Register of Systematic Reviews (PROSPERO) database (https://www.crd.york.ac.uk/prospero) under the number CRD4202020205057. This study was conducted based on the guidelines of the Preferred Reporting Items for Systematic Reviews and Meta-Analysis (PRISMA).

### 2.2. Eligibility Criteria

The research question was established using the acronym “PICO,” where the letter “P” corresponds to adult patients with PXB, “I” to EMGs, “C” to adult patients without PXB, and “O” the effects on the electromyographic activity of the masticatory muscles. Only studies evaluating the electromyographic activity of masticatory muscles in adult patients with PXB were included from retrospective and prospective studies.

Studies involving patients with congenital anomalies and/or syndromes with changes in growth and in craniofacial development were excluded. Animal studies, in-vitro studies, abstracts, and interviews were also excluded.

### 2.3. Search Strategy

The systematic search was carried out by two researchers (LMOG and MCQ) independently. No restrictions have been applied regarding the language and year of publication. The search was conducted between August 22 and September 09, 2020.

### 2.4. Main Search Strategy

Seven electronic databases were searched: MEDLINE/PubMed, EMBASE, Web of Science, Cochrane Library (CENTRAL), SciELO, LILACS, and Scopus.

The search strategy for PubMed used index terms from the Medical Subject Headings (MeSH) and their synonyms. For EMBASE, the index terms from Emtree terms and their synonyms were used.

For the other databases, Cochrane Library, Web of Science, LILACS, SCIELO, and Scopus, combinations of nonindex terms and their synonyms were used. Boolean operators “OR” and “AND” were used for the combination of terms in each database searched (Tables 1).

### 2.5. Additional Search Strategy

An additional search was performed on Google Scholar, Clinical Trials, and gray literature to find unpublished studies or studies published in nonindexed journals. The following terms were used in the search field: “posterior crossbite AND electromyography”.

After the inclusion of the papers, the bibliographical references of the papers were evaluated in an attempt to find studies not located in the places previously mentioned.

### 2.6. Study Selection

The study selection process was performed after the search results were imported into the EndNote X5.0 reference management program for Windows (Clarivate Analytics, https://clarivate.com/). Duplicate papers—found in more than one database—were deleted; only one of them was kept.

The selection process of studies involved two stages. The studies were first selected based on the title and abstract. Noneligible papers were excluded. The papers that passed the first selection had their eligibility evaluated by full reading. Papers that did not meet the inclusion criteria were excluded. The selection process of the studies was carried out by two researchers (LMOG and MCQ) independently. A senior researcher (RRB) was recruited when there was disagreement between the first two.

### 2.7. Data Extraction

Data extraction was carried out by two researchers (LMOG and MCQ) independently. A senior researcher (RRB) was recruited when there was disagreement between the first two. If there were remaining questions, the author of the study would be contacted by e-mail. The following data were extracted from the papers: name of the first author, year of publication, study design, sample characteristics (age, gender, classification of PXB, presence or absence of control group), evaluated muscles, tests performed, electromyograph used, electromyographic parameters, and conclusion of the studies. The data were tabulated and organized using a standard form in software (Excel, Microsoft Office® for Windows—Microsoft®, Redmond, USA).

### 2.8. Evaluation of Study Quality and Risk of Bias

The methodological quality of the studies was independently evaluated by the researchers (LMOG and MCQ) using two evaluation tools.

The first tool used was described by Andrade et al. [20]. The criteria used for the evaluation took into account 8 topics: the study design (randomized clinical trial, controlled clinical trial = 3 points; clinical trial = 1 point); sample size calculation (performed = 1 point); adequate description of the participants' selection method (performed = 1 point); data collection method (performed = 1 point); information regarding the blinding of participants and researchers (informed = 1 point); study of error (performed = 1 point); methodological and statistical analysis (adequate = 1 point); and analysis of confounders (performed = 1 point). The final score of each paper was obtained through arithmetic mean of the sum of the score attributed by the two researchers, where they were classified as low (scores of 0 ≤ 5), average (scores of 5< *x* ≤ 8), and high quality (scores of 8< *x* ≤ 10). If there was any doubt between the two researchers, the study would be discussed with the senior researcher (RRB).

The methodological quality of the studies was also evaluated using the Joanna Briggs Institute (JBI) evaluation tool for systematic reviews [21]. The JBI evaluation consists of the application of an evaluation questionnaire on the risk of bias of the selected studies. Three questionnaires, with eight to nine questions, were applied for each type of study: a questionnaire for cross-sectional studies, a questionnaire for case reports, and a questionnaire for quasiexperimental clinical trials. The possible answers to the questions are “yes,” “no,” “uncertain,” and “not applicable.” The risk of bias of the selected studies was determined based on the number of “yes” responses. Studies with up to 49% of “yes” responses were classified as having high risk of bias; average for studies with 50 to 69% of “yes” responses; and low for studies above 70% of “yes” responses.

The level of agreement between the two researchers was tested by the kappa (*k*) test for categorical variables and by the intraclass correlation coefficient (ICC) for continuous variables [22, 23].

## 3. Results

In total, 6897 records were located searching indexed journals databases and 60 in other sources, adding 6957 records. Of this total, 2401 were excluded because they were duplicated.

**Figure 1:**
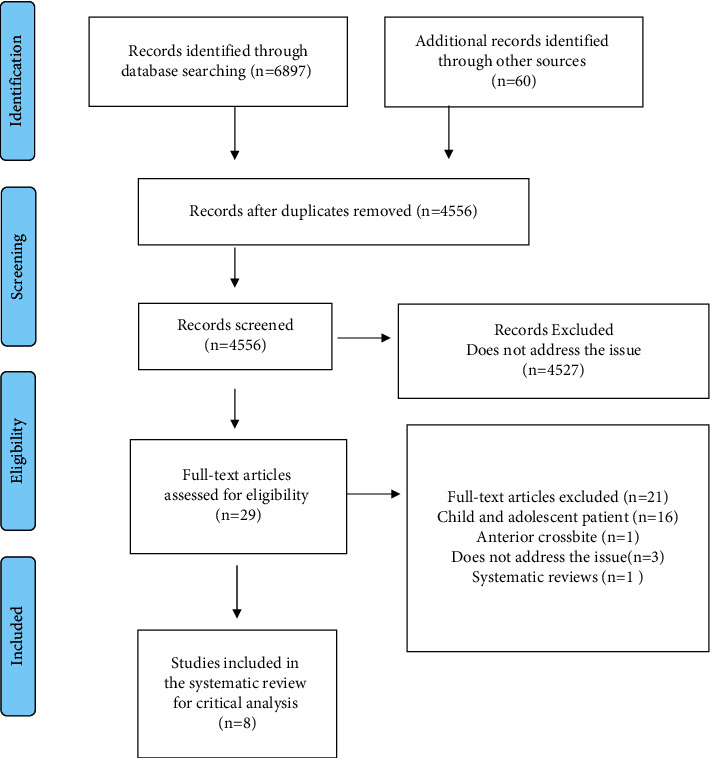
PRISMA flow diagram of study selection.

After removing the duplicates, 4556 records were evaluated for title and abstract. In the first stage, 4527 records were excluded because they did not address the topic of the present study. Then, 29 records were evaluated by reading the full text. In the second stage, 21 papers were excluded: 16 studies with children, one study in patients with anterior crossbite, and three papers that do not address the problem. A systematic review was excluded because it reviewed work included in this review. In the end, eight papers were included in the systematic review for critical analysis: one case report, one quasiexperimental clinical trial, four cross-sectional studies with control group, and two observational studies (Figure 1, Tables 2 and 3).

The selected studies were not analyzed using a meta-analysis, considering the high methodological heterogeneity and the lack of standardized criteria among the different studies, which made it difficult to compare the results.

### 3.1. Studies Details

The selected studies were published between 2003 and 2016. In these publications, 53% of the sample was male. The sample size ranged from 1 to 50 individuals for the PXB group and from 15 to 100 for the control group. In total, 180 individuals with PXB and 180 individuals in the control group were evaluated. Among the selected studies, 62% presented a control group.

Based on the available data, the mean age between the experimental group (21.16 years) and the control group (22.65 years) was similar.

Regarding sample recruitment, patients, students, and volunteers were selected from dental care clinics affiliated with university centers or private clinics. Although all selected studies involved adult individuals with PXB, the presence of PXB was a criterion for inclusion in six studies (75%). Some studies included individuals with other malocclusions, associated or not with PXB [15–18, 42, 43]. The muscles submitted to electromyographic evaluation were in order of higher frequency: superficial masseter (SM), anterior temporal (AT), and posterior temporal (PT) and, the less frequent, sternocleidomastoid (SC), posterior cervical (PC), suprahyoid (SH), upper trapezius (UT), lower trapezius (LT), and anterior digastric belly (DG). While the SM muscle was evaluated in all studies, some muscles such as SH, DG, LT, and PC were evaluated by one study only.

A great variability of equipment was used in the evaluation of electromyographic activity, all of which were of different trademarks. The tests applied in the electromyographic evaluation involved the recording of muscle activity during rest; uni- and bilateral dental tightening; chewing of certain types of food; swallowing; shoulder lifting and lowering movements; flexion and extension movements of the head and neck; besides during mandibular movements. Saifuddin et al. [16] also recorded muscle activity during sleep and during daytime.

The acquisition, filtering, and processing of the electromyographic signal was performed according to the equipment used and according to the methodology used in each study. EMG signals were acquired at a sampling rate ranging from 1000 Hz to 2048 Hz and filtered with a low-pass and high-pass filter ranging from 500 Hz to 10 Hz. The electromyography (EMG) parameters evaluated among the studies were root mean square (RMS) calculation, asymmetry index (AI), torque coefficient (TC), masticatory cycles frequency index (MPF), and electromyographic signal amplitude.

### 3.2. Results for the Evaluation of Study Quality and Risk of Bias

All studies were classified as average to low methodological quality, according to the method described by Andrade et al. [20]. No study presented high quality (Table 4). The calculation of sample size, blinding, study of error, and confounding analysis were the main domains that were not evaluated or considered among the studies.

The evaluated studies with the JBI tool presented moderate to high risk of bias. In the evaluation of the risk of bias for cross-sectional studies, some information was not adequately reported on the description of the sample and on the standardization in the measurement of the condition.

In addition, the identification of confounding factors and the measurement of results were not evaluated in a valid and reliable manner.

The quasiexperimental clinical trial presented problems in the description of the following domains: little information about the cause and effects evaluated, absence of control group, insufficient follow-up time, results measured in an unreliable manner, and inappropriate statistical analysis.

The case report did not adequately describe some information about the methods and procedures related to electromyographic evaluation. In addition, possible adverse effects were not reported (Tables 5–7).

The level of agreement between the two researchers was *K* = 0.925 and ICC = 0.8, which represent, respectively, excellent and good interexaminer agreement [22, 23].

## 4. Discussion

The present study sought to evaluate the literature on the electromyographic evaluation of masticatory muscles in adults with PXB using the PICO strategy, the PRISMA protocol, and the PROSPERO registration. In total, seven electronic databases were searched, from which 8 papers were selected in accordance with the inclusion and exclusion criteria.

To date, only three systematic reviews that evaluated electromyographic activity of masticatory muscles in children, adolescents, and adults with PXB are available in the literature [4, 20, 38]. Only one study conducted in adult patients [4]. These reviews present as limitations the fact that they do not use a comprehensive search strategy, restricted to only a few databases. In addition, the absence of registration and protocol for these reviews reduces its transparency and reproducibility.

The evaluation of the methodological quality in the present search was carried out using two tools. The first proposed by Andrade et al. [20], and the second proposed by the Joanna Briggs Institute (JBI) [21]. The first was performed to compare the results of the present review with those conducted by Andrade et al. [20], Tsanidis et al. [38], and Iodice et al. [4], once all used this evaluation tool.

The studies were classified as average to low methodological quality, similar to those found by those reviews. The second was performed to identify the risk of bias from different study designs and types, evaluating the evidence available from cross-sectional, observational studies, quasiexperimental clinical trials, and case reports [19]. The studies presented moderate to high risk of bias.

### 4.1. Comparison between Individuals with and without PXB

Dong et al. [15] and Saifuddin et al. [16] point out that adults with PXB presented a decrease in the electromyographic activity of the SM muscle independent of the applied test.

According to Woźniak et al. [17], SM and AT muscles showed a decrease in electromyographic activity only during maximum voluntary contraction (MVC). At rest, only SM presented a decrease in electromyographic activity.

Tecco et al. [7] found different results. According to the authors, the AT and SC muscles presented an increase in their electromyographic activity, while in the SM evaluation, they did not find any difference among patients with and without PXB both during MVC and at rest. The difference in results can be attributed to the protocols and to the EMG parameters used.

Among the studies that compared individuals with and without PXB, Tecco et al. [7] did not normalize the muscular electric potential during the electromyographic evaluation stage.

Normalization of the EMG signal is of paramount importance to eliminate possible differences related to bioimpedance, electrode positioning, and muscle morphology.

According to Ferrario et al. [5], the normalization of the EMG signal represents the percentage fraction of the electromyographic record between two measurements: the first in MVC on cotton rolls and the second in usual maximum intercuspation (UMI) without the cotton rolls. In this way, it is possible to normalize the data by the MVC. Thus, the normalization of the EMG signal allows for a more accurate comparison of intra- and interindividual differences.

According to Yamasaki et al. [42], patients with PXB present an altered level of concordance between the preferred side of the bite and the side on which the patient was instructed to chew when evaluated with sEMG. These results reinforce the idea that patients with PXB present changes in electromyographic potentials when compared with individuals without PXB.

Thus, the findings related to the comparison between individuals with and without PXB confirm our initial hypothesis since several studies have found changes in electromyographic activity due to the presence of PXB.

### 4.2. Comparison between Crossbite and Noncrossbite Sides in Adults with UPXB

Dong et al. [15] and Saifuddin et al. [16] evaluated adult patients with UPXB and skeletal mandibular asymmetry. According to Dong et al. [15], these patients present an imbalance in the contraction pattern in SM, SH, SC, and UT muscles between the crossbite and noncrossbite sides, both during MVC and for flexion-extension movements of the head and neck and shoulder elevation-lowering.

These authors observed that while SM and SH muscles presented higher electromyographic activity on the crossbite side, the SC and TR muscles presented lower activity. According to Saifuddin et al. [16], these patients present a lower electromyographic activity of the AT muscle only on the crossbite side.

Tecco et al. [7] and Woźniak et al. [17] observed, in turn, that adult patients with UPXB presented higher electromyographic activity on the crossbite side, mainly in the AT muscle at rest. During MVC, the authors did not find a statistically significant difference.

Moreno et al. [18] found similar results regarding higher electromyographic activity of the AT muscle, however, during MVC and not at rest.

Regarding the electromyographic parameters evaluated, Woźniak et al. [17] observed that patients with PXB presented a significant increase in AI and TC, caused by an imbalance in electromyographic activity between the right and left SM and AT muscles.

Dong et al. [15] found similar results when comparing MPF values that were found to be decreased in these patients. As for RMS, Dong et al. [15] observed that patients with UPXB presented lower rates for the SM muscle on the crossbite side when compared with the noncrossbite side. According to the authors, this result is related to a lower potential for action of the evaluated muscle, pointing to an imbalance in electromyographic activity between the sides.

Although they were measured in a valid way, differences in the evaluation protocols and in the electromyographic evaluation tests may justify the results found. According to Ferrario et al. [5], standardized protocols in electromyographic evaluation are fundamental to minimize possible biases and errors, not only in the capturing but also in the processing of the EMG signal. Although sEMG is a simple diagnostic exam, it is extremely sensitive [5, 34]. In addition, little information was reported on the examiner's experience and on the reliability of the results.

The difference in results can also be attributed to the compensatory adaptive capacity that some individuals develop about electromyographic activity. It should also be considered that some degree of asymmetry in muscle activity in patients with PXB is considered normal and compatible with balanced functional occlusion, as small physiological variations intra- and interindividuals are expected [43].

Thus, the findings related to the comparison between the crossbite side and the noncrossbite side in adults with UPXB partially confirm our initial hypothesis, as several studies have found a reduction in electromyographic activity on the side affected by UPXB.

### 4.3. Changes in Electromyographic Activity after PXB Correction

To increase the clinical applicability of our findings, our review also sought to address the effects of PXB correction on the electromyographic activity of masticatory muscles.

Sverzut et al. [40] and Takeshita et al. [41] evaluated the changes in the electromyographic activity of the masticatory muscles after PXB correction. According to Sverzut et al. [40], the surgically assisted rapid maxillary expansion (SARME) decreased the electromyographic activity in the AT and SM muscles after 15 days of surgery. According to Takeshita et al. [41], the combination of orthosurgical treatment balanced the electromyographic activity of the SM and AT muscle pairs.

The result presented by Sverzut et al. [40] should be evaluated with caution, as the follow-up period was very short. In addition, it is expected that, after 15 days of SARME, patients present some postoperative sensitivity and discomfort, which may make it difficult to perform a reliable electromyographic evaluation.

However well described, the case report presented by Takeshita et al. [41] lacks reliable evidence about the outcomes evaluated, in a way that the results of this study cannot be extrapolated.

### 4.4. Summary of Evidence

Different results were found among the selected papers. Despite the differences, the best available evidence suggests that adult patients with PXB have electromyographic activity changes in the masticatory muscles when compared with individuals without PXB [7, 15–17]. Moreover, adult patients with UPXB have asymmetrical electromyographic activity when the crossbite side is compared with the noncrossbite side.

From a functional point of view, these changes are associated with muscle strength changes. From the electromyographic point of view, these changes are associated with a lower or higher motor units recruited in the time domain.

When evaluated in the area of the trigger excitation frequency, patients with PXB present changes in muscle precision and specificity. As a result, they clinically present an imbalance in masticatory activity between the sides.

### 4.5. Limitations

The present systematic review presents some limitations. Most studies were classified as having average to low methodological quality and moderate to high risk of bias. No study presented high quality and low risk of bias. Among the evaluated domains, the selection of participants, the measurement of outcomes, and the control of confounding factors presented a high risk of bias.

The number of papers and the sample size of the studies that evaluated electromyographic changes in the masticatory muscles in adult patients with PXB were low. In all the studies, there was neither blinding nor randomization of the selected sample. No information regarding sample calculation and statistical power was described in the studies.

Most of the reviewed studies lack information about possible confounding factors. The number of teeth present, the presence of parafunctional habits, associated neuromuscular changes, and facial type were not reported and analyzed. The identification of these factors should be observed to minimize any differences among the groups, as they may influence the direction of the results.

This also applies to differences in amplitude and mean standard deviation of electromyographic potentials in some studies. Large variations affect the homogeneity of variance among the groups, which compromises the comparison among them.

The absence of follow-up and long-term follow-up also represent important biases in the results presented. Finally, different protocols in electromyographic evaluation were used, making it difficult to compare the results.

### 4.6. Recommendations for Future Research

There is a need to improve the quality of evidence on the subject. Studies with longer follow-up time should be conducted, based on a rigorous scientific methodology of preference through clinical trials with a control group and complete description of the sample studied (age, gender, type of malocclusion).

Sample size calculation and the error study should be performed to establish adequate statistical power for the study.

The measurement of the condition in patients with PXB should be performed using well-defined diagnostic criteria and calibrated among the examiners. The same applies to the data analysis, which must be performed in a valid and reliable manner.

In addition, the reproducibility of the measurements and electromyographic parameters should be tested and calibrated among the examiners. Similarly, possible confounding factors should be evaluated and identified to minimize any influence on the evaluated results.

## 5. Conclusion

The results of the present systematic review suggest that adult patients with PXB have electromyographic activity changes in masticatory muscles when compared with individuals without PXB. Moreover, adult patients with UPXB have asymmetrical electromyographic activity when the crossbite side is compared with the noncrossbite side.

These results should be evaluated with some caution, considering the low-average methodological quality and high risk of bias in some domains. Despite the limitations presented, the electromyographic evaluation of masticatory muscles should be considered in the diagnosis and orthodontic treatment plan of patients with PXB, given the impact these changes have on the development and functioning of the masticatory system.

## Figures and Tables

**Table 1:** Search Strategies.

Database	Search strategies
PubMed	Population/Problem
	#1 “Malocclusion” [Mesh] OR “Malocclusion” OR “malocclusions” OR “Crossbite” OR “Crossbites” OR “Cross bite” OR “Bite, Cross” OR “Bites, Cross” OR “Cross Bites” OR “cross-bite” OR “crossbite” OR “posterior cross-bite” OR “crossbite” OR “posterior crossbite” OR “posterior cross bite” OR “posterior cross-bite” OR “unilateral posterior crossbite” OR “unilateral posterior cross bite” OR “unilateral posterior cross-bite” OR “maxillomandibular asymmetry” OR “bite force” OR “inter-arch dental relationship” OR “mandibular shift” OR “lateral shift” OR “laterognathism” OR “laterognathia” OR “mandibular laterognathism” OR “maxillary expansion” OR “rapid maxillary expansion” OR “maxillary transverse discrepancy” OR “transverse discrepancy” OR “orthopedic treatment” OR “orthopedics treatments” OR “rapid palatal expansion” OR “palatal deficiency” OR “bite force” OR “bite forces” OR “Force, Bite” OR “Forces, Bite” OR “Occlusal Force” OR “Force, Occlusal” OR “Forces, Occlusal” OR “Occlusal Forces” OR “Masticatory Force” OR “Force, Masticatory” OR “Forces, Masticatory” OR “Masticatory Forces” OR “Bite Force”[Mesh]
	Intervention
	#2 “Electromyography”[Mesh] OR “Electromyography” OR “Electromyographies” OR “Surface Electromyography” OR “Electromyographies, Surface” OR “Electromyography, Surface” OR “Surface Electromyographies” OR “Electromyogram” OR “Electromyograms” OR “muscle activity” OR “muscles activities” OR “muscle function” OR “muscle functions” OR “electromyographic activity” OR “electromyographical data” OR “electromyographic signals” OR “electromyographical activity” OR “electromyographic examination”
	Outcome
	#3 “Temporal Muscle”[Mesh] OR “Temporal Muscle” OR “Muscle, Temporal” OR “Muscles, Temporal” OR “Temporal Muscles” OR “Masseter Muscle”[Mesh] OR Masseter Muscles OR “Muscle, Masseter” OR “Muscles, Masseter” OR “muscle activity” OR “muscles activities” OR “muscle function” OR “muscle functions” OR “jaw muscle” OR “jaw muscles” OR “Masticatory Muscles”[Mesh] OR “Masticatory Muscles” OR “Masticatory Muscle” OR “Muscle, Masticatory” OR “Muscles, Masticatory” OR “Mastication”[Mesh] OR “Mastication” OR “Chewing” OR “Neck Muscles”[Mesh] OR “Neck Muscles” OR “Muscle, Neck” OR “Muscles, Neck” OR “Neck Muscle” OR “Sternocleidomastoideus” OR “sternocleidomastoideus muscle”
Final search	#1 AND #2 AND #3
Database	Search strategies
EMBASE	Population/Problem
	#1 “crossbite”/exp OR “crossbite” OR “cross bite” OR “malocclusion”/exp OR “malocclusion” OR “dental malocclusion” OR “jaw malocclusion” OR “jaw occlusion disorder” OR “occlusion disorder, jaw” OR “occlusion, mal” OR “tooth malocclusion” OR “posterior crossbite” OR “posterior cross bite” OR “posterior cross-bite” OR “unilateral posterior crossbite” OR “unilateral posterior cross bite” OR “unilateral posterior cross-bite” OR “palatal expansion”/exp OR “palatal expansion” OR “palatal expansion procedure” OR “palatal expansion technique” OR “palatal expander”/exp OR “palatal expander” OR “mandibular asymmetry”/exp OR “bite force” OR “chewing” OR “mastication” OR “mastication force” OR “masticatory apparatus” OR “laterognathia”/exp OR “laterognathia” OR “maxillary expansion”/exp OR “maxillary expansion” OR “rapid palatal expansion”/exp OR “chewing” OR “occlusal force”/exp OR “occlusal force” OR “masticatory force” OR “force, mastication”
	Intervention
	#2 “electromyography”/exp OR “electromyography” OR “electrical myography” OR “electro myography” OR “electro-myographic measurement” OR “electromyographic measurement” OR “myography, electric” OR “quantitative electromyography” OR “surface electromyography” OR “electromyogram”/exp OR “electromyogram” OR “e.m.g.” OR “electric myogram” OR “electrical myogram” OR “electro myogram” OR “EMG” OR “emg activity” OR “evoked emg” OR “electric myography” OR “myography, electric” OR “muscle activity monitor”/exp OR “muscle activity monitor” OR “muscle contraction”/exp OR “muscle contraction” OR “contraction, muscle” OR “muscle action” OR “muscle activity” OR “muscle contracting activity” OR “muscle contraction recording” OR “muscle reaction” OR “muscle shortening” OR “muscular activity” OR “muscular contraction” OR “muscular contraction recording” OR “myocontraction” OR “recording, muscle contraction” OR “skeletal muscle contraction”
	Outcome
	#3 “masseter muscle”/exp OR “masseter muscle” OR “muscle, masseter” OR “musculus masseter” OR “temporalis muscle”/exp OR “temporalis muscle” OR “muscle, temporalis” OR “musculus temporalis” OR “temporal muscle” OR “temporalis muscle transfer” OR “transfer, temporalis muscle” OR “masticatory muscle”/exp OR “masticatory muscle” OR “mastication muscle” OR “masticatory muscles” OR “masticatory musculature” OR “muscle of mastication” OR “muscles of mastication” OR “musculi masticatorii” OR “musculus masticatorius” OR “mastication”/exp OR “mastication”/exp OR “mastication” OR “neck muscle”/exp OR “neck muscle” OR “cervical muscle” OR “muscle, neck” OR “muscle, nuchal” OR “neck muscles” OR “nuchal muscle” OR “sternocleidomastoid muscle”/exp OR “sternocleidomastoid muscle” OR “muscle, sternocleidomastoid” OR “musculus sternocleidomastoideus” OR “sternocleidomastoideus muscle” OR “sternomastoid muscle”
Final search	#1 AND #2 AND #3
Database	Search strategies
Web of science	Population/Problem
	#1 AB = (“Malocclusion” OR “malocclusions” OR “Crossbite” OR “Crossbites” OR “cross Bite” OR “Bite, Cross” OR “Bites, Cross” OR “Cross Bites” OR “cross-bite” OR “crossbite” OR “posterior cross-bite” OR “crossbite” OR “posterior crossbite” OR “posterior crossbite” OR “posterior cross-bite” OR “unilateral posterior crossbite” OR “unilateral posterior cross bite” OR “unilateral posterior cross-bite” OR “maxillomandibular asymmetry” OR “bite force” OR “inter-arch dental relationship” OR “mandibular shift” OR “lateral shift” OR “laterognathism” OR “laterognathia” OR “mandibular laterognathism” OR “maxillary expansion” OR “rapid maxillary expansion” OR “maxillary transverse discrepancy” OR “transverse discrepancy” OR “orthopedic treatment” OR “orthopedics treatments” OR “rapid palatal expansion” OR “palatal deficiency” OR “bite force” OR “bite forces” OR “Force, Bite” OR “Forces, Bite” OR “Occlusal Force” OR “Force, Occlusal” OR “Forces, Occlusal” OR “Occlusal Forces” OR “Masticatory Force” OR “Force, Masticatory” OR “Forces, Masticatory” OR “Masticatory Forces”) OR
	TI = (“Malocclusion” OR “malocclusions” OR “crossbite” OR “Crossbites” OR “Cross Bite” OR “Bite, Cross” OR “Bites, Cross” OR “Cross Bites” OR “cross-bite” OR “crossbite” OR “posterior cross-bite” OR “crossbite” OR “posterior crossbite” OR “posterior cross bite” OR “posterior cross-bite” OR “unilateral posterior crossbite” OR “unilateral posterior cross bite” OR “unilateral posterior cross-bite” OR “maxillomandibular asymmetry” OR “bite force” OR “inter-arch dental relationship” OR “mandibular shift” OR “lateral shift” OR “laterognathism” OR “laterognathia” OR “mandibular laterognathism” OR “maxillary expansion” OR “rapid maxillary expansion” OR “maxillary transverse discrepancy” OR “transverse discrepancy” OR “orthopedic treatment” OR “orthopedics treatments” OR “rapid palatal expansion” OR “palatal deficiency” OR “bite force” OR “bite forces” OR “Force, Bite” OR “Forces, Bite” OR “Occlusal Force” OR “Force, Occlusal” OR “Forces, Occlusal” OR “Occlusal Forces” OR “Masticatory Force” OR “Force, Masticatory” OR “Forces, Masticatory” OR “Masticatory Forces”)
	Intervention
	#2 AB = (“Electromyography” OR “Electromyographies” OR “Surface Electromyography” OR “Electromyographies, Surface” OR “Electromyography, Surface” OR “surface Electromyographies” OR “Electromyogram” OR “Electromyograms” OR “muscle activity” OR “muscles activities” OR “muscle function” OR “muscle functions” OR “electromyographic activity” OR “electromyographical data” OR “electromyographic signals” OR “electromyographical activity” OR “electromyographic examination”) OR
	TI = (“Electromyography” OR “Electromyographies” OR “Surface Electromyography” OR “Electromyographies, Surface” OR “Electromyography, Surface” OR “Surface Electromyographies” OR “Electromyogram” OR “Electromyograms” OR “muscle activity” OR “muscles activities” OR “muscle function” OR “muscle functions” OR “electromyographic activity” OR “electromyographical data” OR “electromyographic signals” OR “electromyographical activity” OR “electromyographic examination”)
	Outcome
	#3 AB = (“Temporal Muscle” OR “Muscle, Temporal” or “Muscles, Temporal” OR “Temporal Muscles” OR Masseter Muscles OR “Muscle, Masseter” OR “Muscles, Masseter” OR “muscle activity” OR “muscles activities” OR “muscle function” OR “muscle functions” OR “jaw muscle” OR “jaw muscles” OR “Masticatory Muscles” OR “Masticatory Muscle” OR “Muscle, Masticatory” OR “Muscles, Masticatory” OR “Mastication” OR “Chewing” OR “Neck Muscles” OR “Muscle, Neck” OR “Muscles, Neck” OR “Neck Muscle” OR “Sternocleidomastoideus” OR “sternocleidomastoideus muscle”) OR
	TI = (“Temporal Muscle” OR “Muscle, Temporal” or “Muscles, Temporal” OR “Temporal Muscles” OR Masseter Muscles OR “Muscle, Masseter” OR “Muscles, Masseter” OR “muscle activity” OR “muscles activities” OR “muscle function” OR “muscle functions” OR “jaw muscle” OR “jaw muscles” OR “Masticatory Muscles” OR “Masticatory Muscle” OR “Muscle, Masticatory” OR “Muscles, Masticatory” OR “Mastication” OR “Chewing” OR “Neck Muscles” OR “Muscle, Neck” OR “Muscles, Neck” OR “Neck Muscle” OR “Sternocleidomastoideus” OR “sternocleidomastoideus muscle”)
Final search	#1 (“AB = “ OR “TI = “) AND #2 (“AB = “ OR “TI = “) AND #3 (“AB = “ OR “TI = “)
Database	Search strategies
Cochrane Library	Population/Problem
	#1 “Malocclusion”[Mesh] OR “Bite Force”[Mesh] OR “Malocclusion” OR “malocclusions” OR “Crossbite” OR “Crossbites” OR “Cross Bite” OR “Bite, Cross” OR “Bites, Cross” OR “Cross Bites” OR “cross-bite” OR “crossbite” OR “posterior cross-bite” OR “crossbite” OR “posterior crossbite” OR “posterior cross bite” OR “posterior cross-bite” OR “unilateral posterior crossbite” OR “unilateral posterior cross bite” OR “unilateral posterior cross-bite” OR “maxillomandibular asymmetry” OR “inter-arch dental relationship” OR “mandibular shift” OR “lateral shift” OR “laterognathism” OR “laterognathia” OR “mandibular laterognathism” OR “maxillary expansion” OR “rapid maxillary expansion” OR “maxillary transverse discrepancy” OR “transverse discrepancy” OR “orthopedic treatment” OR “orthopedics treatments” OR “rapid palatal expansion” OR “palatal deficiency” OR “bite force” OR “bite forces” OR “force, bite” OR “forces, bite” OR “occlusal force” OR “force, occlusal” OR “forces, occlusal” OR “occlusal forces” OR “masticatory force” OR “force, Masticatory” OR “forces, masticatory” OR “masticatory forces”
	Intervention
	#2 “Electromyography”[Mesh] OR “Electromyography” OR “Electromyographies” OR “Surface Electromyography” OR “Electromyographies, Surface” OR “Electromyography, Surface” OR “surface Electromyographies” OR “Electromyogram” OR “Electromyograms” OR “muscle activity” OR “muscles activities” OR “muscle function” OR “muscle functions” OR “electromyographic activity” OR “electromyographical data” OR “electromyographic signals” OR “electromyographical activity” OR “electromyographic examination”
	Outcome
	#3 “Temporal Muscle”[Mesh] OR “Masseter Muscle”[Mesh] OR “Masticatory Muscles”[Mesh] OR “Mastication”[Mesh] OR “Neck Muscles”[Mesh] OR “Masseter” OR “Temporal Muscle” OR “Muscle, Temporal” OR “Muscles, Temporal” OR “Temporal Muscles” OR Muscles OR “Muscle, Masseter” OR “Muscles, Masseter” OR “muscle activity” OR “muscles activities” OR “muscle function” OR “muscle functions” OR “jaw muscle” OR “jaw muscles” OR “Masticatory Muscles” OR “Masticatory Muscle” OR “Muscle, Masticatory” OR “Muscles, Masticatory” OR “Mastication” OR “Chewing” OR “Neck Muscles” OR “Muscle, Neck” OR “Muscles, Neck” OR “Neck Muscle” OR “Sternocleidomastoideus” OR “sternocleidomastoideus muscle”
Final search	#1 AND #2 AND #3
Database	Search strategies
SciELO	Population/Problem
	#1 (posterior crossbite)
	Intervention
	#2 (electromyography)
	Outcome
	#3 (temporal OR masseter OR sternocleidomastoid)
Final search	#1 AND #2 AND #3
Database	Search strategies
Lilacs	Population/Problem
	#1 (“Crossbite” OR “Crossbite-group” OR “Crossbite-side” OR “Crossbite/” OR “Crossbites” OR “Crossbites/”)
	Intervention
	#2 (“Electromyograph” OR “Electromyographic” OR “Electromyographicactivity” OR “Electromyographical” OR “Electromyographicdata” OR “Electromyographicrecordings” OR “Electromyographics” OR “Electromyographicsignal” OR “Electromyographie” OR “Electromyographies” OR “Electromyographies, Surface” OR “Electromyographies, Surface/” OR “Electromyographies/” OR “Electromyographique” OR “Electromyographiques” OR “Electromyographs” OR “Electromyography” OR “Electromyography feedback” OR “Electromyography feedback/” OR “Electromyography's” OR “Electromyography, Surface” OR “Electromyography, surface/” OR “Electromyography-Emg” OR “Electromyography/” OR “Electromyography/methods” OR “Electromyography/utilization” OR “Electromyographyc-biofeedback” OR “Electromyographymethods” OR “Electromyographyresults”)

	Outcome
	#3 ((“TempORal muscle” OR “TempORal muscle/” OR “TempORal muscles” OR “TempORal muscles/”) OR (“Masseter muscle” OR “Masseter muscle/” OR “Masseter muscles/” OR “Masseter” OR “Masseter-” OR “Masseter/”) OR (“Sternocleidomastoide” OR “Sternocleidomastoideus” OR “Sternoclidomastoid” OR “Sternomaistoideu” OR “Sternomastoideus” OR “Sternoscleidomastoide”))
Final search	#1 AND #2 AND #3
Database	Search strategies
Scopus	Population/Problem
	#1 “Malocclusions” OR “Crossbite” OR “Crossbites” OR “Cross Bite” OR “Bite, Cross” OR “Bites, Cross” OR “Cross Bites” OR “cross-bite” OR “crossbite” OR “posterior cross-bite” OR “crossbite” OR “posterior crossbite” OR “posterior cross-bite” OR “unilateral posterior crossbite” OR “unilateral posterior crossbite” OR “unilateral posterior cross-bite” OR “maxillomandibular asymmetry” OR “inter-arch dental relationship” OR “mandibular shift” OR “lateral shift” OR “maxillary expansion” OR “Rapid maxillary expansion” OR “maxillary transverse discrepancy” OR “transverse discrepancy” OR “orthopedic treatment” OR “orthopedics treatments” OR “rapid palatal expansion” OR “palatal deficiency” OR “Bite Force” OR “Bite Forces” OR “Force, Bite” OR “Forces, Bite” OR “Occlusal Force” OR “Force, Occlusal” OR “Forces, Occlusal” OR “Occlusal Forces” OR “Masticatory Force” OR “Force, Masticatory” OR “Forces, Masticatory” OR “Masticatory Forces”
	Intervention
	#2 “Electromyography” OR “Electromyographies” OR “Surface Electromyography” OR “Electromyographies, Surface” OR “Electromyography, Surface” OR “Surface Electromyographies” OR “Electromyogram” OR “Electromyograms” OR “muscle activity” OR “muscles activities” OR “muscle function” OR “muscle functions” OR “electromyographic activity” OR “electromyographical data” OR “electromyographic signals” OR “electromyographical activity” OR “electromyographic examination”
	Outcome
	#3 “Temporal Muscle” OR “Muscle, Temporal” or “Muscles, Temporal” OR “Temporal Muscles” OR “Masseter Muscle” OR Masseter Muscles OR “Muscle, Masseter” OR “Muscles, Masseter” OR “muscle activity” OR “muscles activities” OR “muscle function” OR “muscle functions” OR “jaw muscle” OR “jaw muscles” OR “Masticatory Muscles” OR “Masticatory Muscle” OR “Muscle, Masticatory” OR “Muscles, Masticatory” OR “Mastication” OR “Chewing” OR “Neck Muscles” OR “Muscle, Neck” OR “Muscles, Neck” OR “Neck Muscle” OR “Sternocleidomastoideus” OR “sternocleidomastoideus muscle”
Final search	#1 AND #2 AND #3

**Table 2:** List of excluded articles listed in alphabetical order of first author, with the principal reason for exclusion.

Author/year	Article title	Reason for exclusion
Alarcón, et al. [24], 1997	Electromyographic activity of masticatory muscles in children with posterior crossbite	Child/adolescent
Alarcón et al. [19], 2000	Effect of unilateral posterior crossbite on the electromyographic activity of human masticatory muscles	Child/adolescent
Alarcón et al. [25], 2009	Activity of jaw muscles in unilateral crossbite without mandibular shift	Child/adolescent
Andrade et al. [20], 2008	Posterior crossbite and functional changes: A systematic review	Child/adolescent
Arat et al. [26], 2008	Muscular and condylar response to rapid maxillary expansion. Part 1: Electromyographic study of anterior temporal and superficial masseter muscles	Child/adolescent
Di Palma et al. [13], 2017	Longitudinal effects of rapid maxillary expansion on masticatory muscles activity	Child/adolescent
Farronato et al. [27], 2012	Rapid palatal expansion: Electromyographic and electrognatographic evaluations	Child/adolescent
Farronato et al. [28], 2012	Electromyographic and electrognatographic evaluations during rapid palatal expansion. A case report	Child/adolescent
Ferrario et al. [6], 1999	The influence of crossbite on the coordinated electromyographic activity of human masticatory muscles during mastication	Child/adolescent
Go [29], 1981	An electromyographic study on masticatory muscles—comparison and examination of crossbite patients preoperatively, postoperatively and in post retention	Anterior crossbite
Handa [30], 1981	A study on the changes of jaw movement and EMG pattern induced by rapid palatal expansion	Child/adolescent
Iodice, et al. [4], 2016	Association between posterior crossbite, skeletal, and muscle asymmetry: A systematic review	Systematic reviews
Kwak et al. [31], 2014	Functional evaluation of orthopedic and orthodontic treatment in a patient with unilateral posterior crossbite and facial asymmetry	Child/adolescent
Maffei et al. [32], 2014	Orthodontic intervention combined with myofunctional therapy increases electromyographic activity of masticatory muscles in patients with skeletal unilateral posterior crossbite	Child/adolescent
Munro [33], 1975	Electromyography of the muscles of mastication	Does not address the issue
Piancino et al. [34], 2016	Effects of therapy on masseter activity and chewing kinematics in patients with unilateral posterior crossbite	Child/adolescent
Piancino et al. [35], 2009	Muscular activation during reverse and non-reverse chewing cycles in unilateral posterior crossbite	Child/adolescent
Regalo et al. [36], 2018	Analysis of the stomatognathic system of children according orthodontic treatment needs	Child/adolescent
Sonnesen and Bakke [37], 2007	Bite force in children with unilateral crossbite before and after orthodontic treatment. A prospective longitudinal study	Does not address the issue
Tsanidis et al. [38], 2016	Functional changes after early treatment of unilateral posterior crossbite associated with mandibular shift: A systematic review	Child/adolescent
Maspero et al. [39], 2012	Functional appliance Andreasen and electromyographic evaluations. A literature review	Does not address the issue

**Table 3:** Characteristics of the included studies.

Author/year	Design	Sample details	Sample age (sd)	Control group	Control age (sd)	Evaluated muscles	Applied tests	Electromyographer	Electromyographic parameters	Conclusion
Dong et al. [15], 2008	Cross-sectional study with control group	*N* = 15 (8F and 7M) UPXB = 9; PXB = 6	23.0 (4.78)	*N* = 21 (10F and 11M)	22.0 (0.92)	SM, SC, SH, ST	Maximum jaw opening and closing, head and neck flexion-extension, shoulder lifting and lowering	K7 Evaluation System: 8 channels	RMS, MPF	Individuals with mandibular asymmetry and PXB are associated with more asymmetric electromyographic patterns
Moreno et al. [18], 2008	Observational study	*N* = 3 UPXB	24	Absent	Absent	SM, AT, PT, DG	Swallowing record (water), chewing record for 15 sec (3 chips), MVC for 3 sec	Myotronics ® K6-i: 8 channels	Signal amplitude (µV)	Individuals with UPXB presented increased electromyographic activity of the AT on the cross-side during MVC
Saifuddin et al. [16], 2003	Cross-sectional study with control group	*N* = 15 (5F and 10M) PXB	19.9 (5.3)	*N* = 15 (2F and 13M)	28.6 (1.9)	SM and AT	Diurnal recording (2 periods: meals + regular activities) and night recording (sleep)	Muscle Tester ME3000P (Mega Electronics Ltd, Kuopio, Finland)	Signal amplitude (µV), asymmetry index	Individuals with mandibular deviation associated to PXB presented lower electromyographic activity in SM and AT muscles during diurnal recordings
Sverzut et al. [40], 2011	Quasiexperimental clinical trial	*N* = 19 (13F and 6M) PXB	25.4	*N* = 19 (13F and 6M)	25.4	SM and AT	Usual chewing (gum), dental tightening, mouth opening and closing, rest, protrusion, and laterality (D and E)	MyoSystem-Br1 (São Paulo, Brazil)	RMS	Electromyographic activity decreases in SM and AT muscles after SARME during chewing and dental tightening
Takeshita et al. [41], 2013	Case report	*N* = 1 (1F); skeletal class III with mandibular asymmetry, UPXB and AXB	18.8	Absent	Absent	SM, AT, and PT	Left and right unilateral chewing and dental tightening	No information	Electromyographic activity µV/s	Orthosurgical treatment determined a balanced and symmetrical chewing muscle activity
Tecco et al. [7], 2010	Cross-sectional study with control group	*N* = 75 (30F and 45M) UPXB: 50; bilateral PXB:25	M: 19.5 (5.6); F: 20.4 (3.2)	*N* = 25 (6F and 19M)	22.5 (5.8)	SM, AT, PT, SC, PC, ST, and LT	Rest and MVC	Key-Win 2.0 (Biotronic s.r.l., San Benedetto Del Tronto, Ascoli Piceno, Italy): 60 channels	Electromyographic activity µV/s	Patients with PXB present electromyographic changes in the chewing muscles, neck, and upper trunk
Woźniak [17], 2015	Cross-sectional study with control group	*N* = 50 (22F and 28M) UPXB and TMD	20.84 (1.14)	*N* = 100 (54F and 46M)	21.42 (1.06)	SM and AT	Rest and MVC	DAB-Bluetooth Instrument (Zebris Medical GmbH, Germany	Asymmetry index (AI) and torque coefficient (TC)	UPXB was associated with changes in SM and AT muscle activity
Yamasaki, et al. [42], 2015	Observational study	*N* = 2 PXB	31.3	Absent	Absent	SM	Left and right unilateral chewing of different types of food (peanut, beef jerk, and chewing gum), and MVC	ProComp INFINITI; Thought Technology, (Montreal, Canada)	RMS	Individuals with PXB present an altered level of agreement regarding the parameters of identification of the preferred side of the bite

F, female gender; M, male gender; PXB, posterior crossbite; UPXB, unilateral posterior crossbite; AXB, anterior crossbite; TMD, temporomandibular disorder; sd: standard deviation; AT, anterior temporal muscle; PT, posterior temporal muscle; SM, superficial masseter muscle; SC, sternocleidomastoid muscle; SH, suprahyoid muscles; UT, upper trapezium muscle; LT, lower trapezius muscle; DG, digastric muscle; PC, posterior cervical muscles; SARME, surgically assisted rapid maxillary expansion; POC, percent overlapping coefficient; RMS, root mean square; MPF, mean power frequency; MVC, maximum voluntary contraction; µV, microvolts; *R*, right; L, left.

**Table 4:** Quality evaluation of the selected studies.

Author/year	Study design	Sample size	Selection description	Valid measurement methods	Blinding in measurements	Method error analysis	Adequate statistics provided	Confounding factors	Judged quality standard
Dong et al. [15], 2008	Cross-sectional studies with control group	No	Adequate	Adequate	No	No	Adequate	No	Medium (6)
Moreno et al. [18], 2008	Observational study	No	Adequate	Adequate	No	No	Adequate	No	Low (4)
Saifuddin et al. [16], 2003	Cross-sectional studies with control group	No	Adequate	Adequate	No	No	Adequate	No	Medium (6)
Sverzut et al. [40], 2011	Quasiexperimental clinical trial	No	Adequate	Adequate	No	No	Adequate	No	Medium (6)
Takeshita et al. [41], 2013	Case report	No	Inadequate	Inadequate	No	No	Not related	No	Low (1)
Tecco et al. [7], 2010	Cross-sectional studies with control group	No	Adequate	Adequate	No	Yes	Adequate	No	Medium (7)
Woźniak [17], 2015	Cross-sectional studies with control group	No	Adequate	Adequate	No	No	Adequate	No	Medium (6)
Yamasaki et al. [42], 2015	Observational study	No	Inadequate	Adequate	No	Yes	Adequate	No	Low (4)

Quality evaluation, according to Andrade et al. [20].

**Table 5:** JBI critical appraisal tool results for analytical cross-sectional studies.

	Were the criteria for inclusion in the sample clearly defined?	Were the study subjects and the setting described in detail?	Was the exposure measured in a valid and reliable way?	Were objective, standard criteria used for measurement of the condition?	Were confounding factors identified?	Were strategies to deal with confounding factors stated?	Were the outcomes measured in valid and reliable way?	Was appropriate statistical analysis used?	% of “yes” scores	Risk of bias
Dong et al. [15], 2008	Yes	Unclear	Unclear	Yes	Unclear	Unclear	Unclear	Yes	37%	High
Moreno et al. [18], 2008	No	No	Yes	Yes	Unclear	Unclear	Yes	Yes	50%	Moderate
Saifuddin et al. [16], 2003	Yes	Unclear	Yes	Yes	Unclear	Unclear	Yes	Yes	62%	Moderate
Tecco et al. [7], 2010	Yes	Yes	Yes	Unclear	Unclear	Unclear	Yes	Yes	62%	Moderate
Woźniak [17], 2015	Yes	Yes	Unclear	No	Unclear	Unclear	Unclear	Yes	37%	High
Yamasaki et al. [42], 2015	Yes	No	Yes	No	Unclear	Unclear	Unclear	Yes	37%	High

**Table 6:** JBI critical appraisal tool result for quasiexperimental study.

	Is it clear in the study what is the “cause” and what is the “effect”?	Were the participants included in any comparisons similar?	Were the participants included in any comparisons receiving similar treatment/care, other than the exposure or intervention of interest?	Was there a control group?	Were there multiple measurements of the outcome both pre and post the intervention/exposure?	Was follow-up complete and if not, were differences between groups in terms of their follow-up adequately described and analyzed?	Were the outcomes of participants included in any comparisons measured in the same way?	Were outcomes measured in a reliable way?	Was appropriate statistical analysis used?	% of “yes” scores	Risk of bias
Sverzut et al. [40], 2011	No	Yes	Yes	No	Yes	Unclear	Yes	No	Unclear	55%	Moderate

**Table 7:** JBI critical appraisal tool result for case report.

	Were patient's demographic characteristics clearly described?	Was the patient's history clearly described and presented as a timeline?	Was the current clinical condition of the patient on presentation clearly described?	Were diagnostic tests or assessment methods and the results clearly described?	Was the intervention(s) or treatment procedure(s) clearly described?	Was the postintervention clinical condition clearly described?	Were adverse events (harms) or unanticipated events identified and described?	Does the case report provide takeaway lessons?	% of “yes “scores	Risk of bias
Takeshita et al. [41], 2013	Yes	Yes	Yes	Unclear	Unclear	Yes	No	Yes	62%	Moderate

## Data Availability

All data are available with the corresponding author and will be released upon request.
